# Lipid Rafts and Caveolae in Signaling by Growth Factor Receptors

**DOI:** 10.2174/1874091X00701010012

**Published:** 2007-09-13

**Authors:** Angela de Laurentiis, Lorna Donovan, Alexandre Arcaro

**Affiliations:** 1 Division of Clinical Chemistry and Biochemistry, University Children’s Hospital Zurich, Steinwiesstrasse 75, CH-8032 Zurich, Switzerland; 2 Division of Medicine, Imperial College Faculty of Medicine, Hammersmith Hospital, Du Cane Road, London W12 ONN, UK

## Abstract

Lipid rafts and caveolae are microdomains of the plasma membrane enriched in sphingolipids and cholesterol, and hence are less fluid than the remainder of the membrane. Caveolae have an invaginated structure, while lipid rafts are flat regions of the membrane. The two types of microdomains have different protein compositions (growth factor receptors and their downstream molecules) suggesting that lipid rafts and caveolae have a role in the regulation of signaling by these receptors. The purpose of this review is to discuss this model, and the implications that it might have regarding a potential role for lipid rafts and caveolae in human cancer. Particular attention will be paid to the epidermal growth factor receptor, for which the largest amount of information is available. It has been proposed that caveolins act as tumor suppressors. The role of lipid rafts is less clear, but they seem to be capable of acting as ‘signaling platforms’, in which signal initiation and propagation can occur efficiently.

## INTRODUCTION

The ‘fluid mosaic’ model envisions the plasma membrane to contain proteins floating freely in a uniform ‘sea’ of lipid. However, the plasma membrane is no longer thought of as a uniform structure, following the identification of microdomains known as lipid rafts and caveolae. These microdomains are of a less fluid character than the remainder of the membrane, and are enriched in certain proteins, in particular those involved in signal transduction. This has led to the hypothesis that they might act as membrane signalling platforms. The aim of this article is to discuss the possible role that these membrane microdomains play in growth factor receptor signal transduction. We will therefore begin by describing the structure of lipid rafts and caveolae and the proteins that are targeted to these domains, before going on to discuss in detail their possible roles in the signalling of certain growth factor receptors. Lastly, we will discuss how the alteration of signalling in these microdomains may lead to cancer in humans.

## CAVEOLAE

**Fig. (1):**
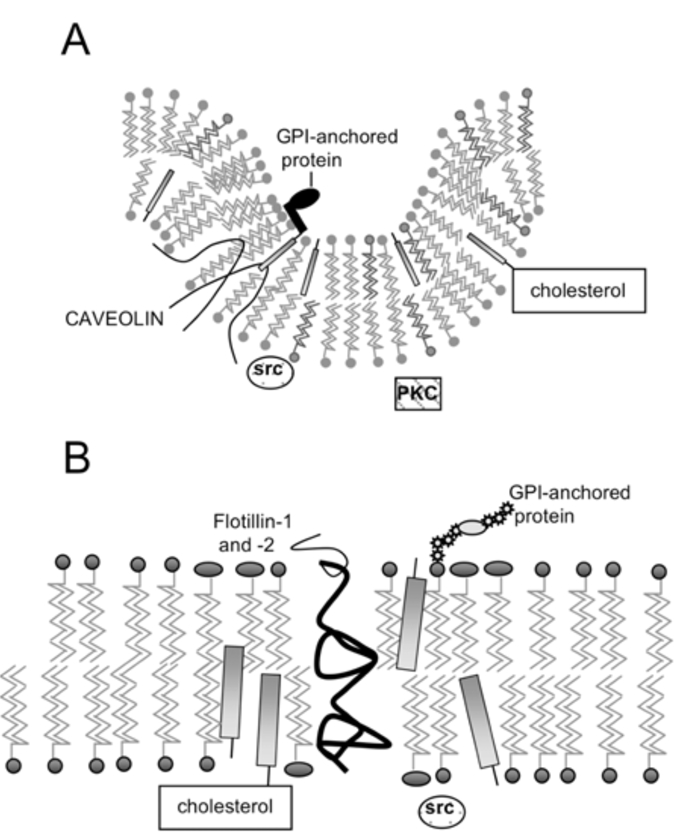
**Structure of lipid rafts and caveolae. A) Caveolae; B) Lipid Rafts.** Caveolin molecules in (**A**) are shown as hairpin-shaped structures in the inner leaflet; N- and C-termini project into the cytoplasm. GPI-anchored proteins are anchored on the outer leaflet of caveolae and lipid rafts, while signalling molecules such as Src kinase associate with the inner leaflet.

Caveolae were first described in the 1950s as 50-100 nm flask-shaped invaginations of the plasma membrane [1] (Fig. 1**A**). Caveolae have been found to be insoluble in non-ionic detergents such as Triton X-100, and this property has been used to purify and characterise these domains. Following treatment with detergent at 4°C, these insoluble domains are then separated from the remainder of the cell membrane by ultracentrifugation in a sucrose density gradient [2]. Alternatively, a detergent-free fractionation procedure can be used, following which caveolae are present in low buoyant density fractions [3]. The insolubility of caveolae in detergent is thought to be due to an enrichment in sphingolipids and cholesterol [4, 5], as opposed to the majority of the cell membrane, which is composed mainly of glycerophospholipids. Unsaturated acyl chains are common in the latter, while sphingolipids contain largely saturated chains, allowing them to pack together more tightly. Hence, glycerophospholipids exist in a loosely packed, liquid-disordered phase (the liquid crystalline, l_c_ phase). In the absence of cholesterol, sphingolipids form a very solid phase known as the gel phase; however, in caveolae cholesterol is present, and this causes these domains to exist in a new phase, the liquid-ordered phase (l_o_) [6]. In this phase the lipids are still tightly packed, but also have a high degree of lateral mobility. These properties confer insolubility in non-ionic detergents, and depletion of these domains of either sphingolipids or cholesterol increases their solubility in Triton X-100 [7].

Caveolae possess a striated coat on their cytoplasmic face, major components of which are proteins known as caveolins. There are now three known members of the cave-olin gene family: caveolin-1 (two isoforms α and β) [8, 9], caveolin-2 (three isoforms α, β and δ) [10] and caveolin-3 [11]. The N- and C-termini of caveolins are cytoplasmic, suggesting that they form a ‘hairpin’ structure in the membrane (Fig. 1**A**), and they are palmitoylated on several residues [12]. Caveolins –1 and –2 have a wide, overlapping distribution: they (and therefore caveolae) are present in most cell types, being most abundant in endothelial cells, fibroblasts, adipocytes, pneumocytes and epithelial cells [13, 14]. In contrast, caveolin-3 is almost solely expressed in smooth and skeletal muscle [15].

Two forms of caveolae, ‘deep’ and ‘shallow’, have been identified, with different distribution of the three caveolins and the respective isoforms [10, 16]. It is possible that these domains have functional differences, but this has not been sufficiently studied. Caveolins are thought to be an important structural component of caveolae, which is probably related to the ability of caveolin to bind cholesterol [17] and sphingolipids [18]. More precisely caveolin-1 or caveolin-3 re-expression in cells lacking both caveolae and caveolin expression induces caveolae formation and reconstitutes the caveolar coat [19]. However, expression of caveolin-2 alone doesn’t lead to caveolae formation, but requires co-transfection of caveolin-1 [19, 20] Caveolins are phosphorylated on Ser/Thr sites by PKCα and on Tyr by Src and possibly other tyrosine kinases [21].

The number of caveolae in a cell and the level of caveolin expression are also intimately linked to the concentration of cholesterol. Increasing the level of free cholesterol in fibroblasts by increasing the external concentration of low density lipoprotein (LDL) led to an upregulation of caveolin mRNA and increased the number of caveolae [22], while depleting cholesterol using cholesterol-sequestering agents led to a decrease in caveolin mRNA levels [23]. In turn, decreasing caveolin concentration lowered the rate of cholesterol efflux from the cells, suggesting that caveolae are involved in cholesterol homeostasis [22].

In addition to caveolins, another family of proteins has been shown to form an integral component of caveolae. These are the flotillins, flotillin-1 and flotillin-2 (epidermal surface antigen) [24]. Flotillins can form hetero-oligomeric complexes with caveolins, and are probably structural components, which participate in the formation of caveolae [25].

## LIPID RAFTS

The discovery that detergent-insoluble domains were present in cells lacking caveolae and caveolins [26, 27] suggested that membrane microdomains distinct from caveolae might exist. These domains can also co-exist with caveolae in the same cell, and can be isolated separately [28] and are present in the same membrane fractions as caveolin, using detergent-dependent or –independent methods. They have been coined ‘lipid rafts’ [29], and have an estimated diameter of less than 70 nm [30], possibly being as little as 26+/-13nm [31]. Like caveolae, they are enriched in spingolipids and cholesterol, but do not seem to form any particular structure as caveolae do, rather being flat regions of the cell membrane that exist in the liquid ordered phase (Fig. **1B**). Unlike caveolae, they are probably found ubiquitously, while caveolae are excluded from certain cells, such as T lymphocytes [32].

There have been concerns that lipid rafts might be artefacts arising from the treatment of membranes with detergent at low temperature [33]. Firstly, treatment with Triton X-100 is usually performed at 4°C, and the cooling of the membrane by itself promotes the formation of the liquid ordered phase [6]. In addition, Triton X-100 was shown to promote the formation of liquid-ordered domains when added to an originally homogeneous membrane [34]. Despite these observations, there is evidence that lipid rafts exist in the liquid ordered phase prior to addition of detergent. Ahmed *et al.* [5] used a detergent-free, fluorescence quenching technique to show that phase separation did occur in membranes with a similar composition to the plasma membrane, suggesting the co-existence of a liquid-ordered phase containing sphingolipids and cholesterol, and a liquid crystalline phase. Phase separation occurred at 37°C, and was promoted by cholesterol. Furthermore, the degree of insolubility in Triton X-100 correlated with the proportion of the membrane that was in the liquid-ordered phase. This suggests that these domains are probably present before detergent extraction; however it must still be noted that detergent probably alters the properties of these domains and their interaction with certain proteins, and that results obtained following treatment of membranes with detergent must be treated with caution. The detergent method certainly cannot be used to determine the difference between lipid rafts and caveolae when both these domains are present in the same cell.

Other lines of evidence also support the existence of lipid rafts in cell membranes [35]. For example, Varma and Mayor [30] used fluorescence resonance energy transfer (FRET) to show that glycosylphosphatidylinositol (GPI)-anchored folate receptors attached to a fluorescent folic acid analogue were clustered in domains of a size less than 70 nm. Depleting the cell membrane of cholesterol resulted in a decreased FRET efficiency, consistent with an increased distance between folate receptors. Addition of cholesterol to the cell resulted in restoration of the proteins to ordered domains. These results have been taken as proof of the existence of cholesterol-rich microdomains into which these proteins cluster. This hypothesis is further supported by work by Friedrichson and Kurzchalia [36], who used chemical cross-linking to demonstrate that GPI-anchored proteins were clustered at the plasma membrane, and that depletion of cholesterol decreased clustering, whereas addition of cholesterol increased the size of the clusters. The strongest evidence for non-caveolae nanodomains (50–200 nm in dimension in the outer leaflet of the plasma membrane) comes from immunogold electron microscopy of components of, for example, the T cell receptor (TCR) in fixed cells, where labelled proteins are detected in clusters [35].

Since lipid rafts are small and transient, the biophysical challenges of measuring them are still great. Indeed, the most controversial area of membrane lateral organization is on the nanoscale level, where technology with sufficient simultaneous spatial and temporal resolution is not yet available. Refining existing methods and developing new ones to study lipid rafts is now required for progress, with the goal of studying dynamic membrane structure in living cells [35]. The applicable techniques, for the most part, rely on fluorescence microscopy because of high sensitivity and applicability to single, living cells. Several techniques with the potential to most directly detect and characterize lipid rafts in living cells were reviewed recently [35].

Interestingly, it has been shown that flotillin-1 and flotillin-2 (also known as reggie-2 and –1) are localised in lipid rafts [37] and co-localise with GPI-anchored cell adhesion molecules, known to be found in rafts. It is necessary to note here that, although the outer leaflet of lipid rafts (Fig. 1) is thought to be composed of sphingolipids and cholesterol, the nature of the inner leaflet in unknown.

## PROTEINS ASSOCIATED WITH LIPID RAFTS AND CAVEOLAE

Besides the caveolins and flotillins, numerous other proteins have been associated with lipid rafts and caveolae. As mentioned above, there is evidence for the clustering of GPI-anchored proteins in lipid rafts, and they have also been identified in caveolae [38]. The existence of different functional GPI anchors as well as the fact that different rafts show markedly different lipid and protein profiles implies the presence of heterogeneous group of anchors and corresponding rafts [39, 40].

In addition, many proteins associated with the microdomains are receptors or proteins involved in signal transduction. These include G protein α-subunits, which have been shown to bind to caveolin *in vitro* [41]. A later study showed that different G protein subtypes localised preferentially to particular microdomains [42]. Several G-protein-coupled receptors have also been localised to membrane microdomains; some of these only become localised to these domains upon activation – for example the muscarinic acetyl choline receptor in caveolae [43]. Interestingly, a downstream component of signalling by this receptor, endothelial nitric oxide synthase, is localised to caveolae, and is thought to be negatively regulated by caveolin [44].

A number of other proteins that are able to interact with caveolin localise to caveolae. A detergent-free method, which gives a more accurate view of the proteins associated with rafts/caveolae than the detergent method [45], was used to demonstrate the co-fractionation of caveolin with H-Ras [46], c-Src, cdc42, Rho, Lyn and Fyn [47]. Ras was found to interact with caveolin, although a mutationally activated form of Ras would not bind to caveolin [48]. Furthermore, another study showed that caveolin binds to c-Src, although it will not bind mutationally-activated v-Src [49]. In addition, binding of caveolin-1 or caveolin-3 inhibited the auto-phosphorylation and activation of c-Src and the related Src-family tyrosine kinase Fyn [49]. These interactions were mediated by residues 82-101 in the cytoplasmic N-terminus of caveolin-1, a region of the protein which is called the ‘scaffolding domain’, and which interacts with numerous proteins. Furthermore, it has been described that caveolin phosphorylated on Tyr 14 can inhibit Src through the recruitment of c-terminal src kinase (Csk). The scaffolding domain of caveolin-1 seems also to be responsable for further interactions, since it contains a motif conserved in guanine nucleotide dissociation inhibitors (GDIs) which suggested a novel role for caveolin-1. Nevins and Thurmond demonstrated the interaction between caveolin-1 and Cdc42 [50]. Further studies also revealed a role in the activation and regulation of Rho, suggesting a role of caveolin-1 in cell polarity and migration [51]. del Pozo *et al*. [52] reported that lipid rafts are involved in signal transduction events initiated by cell adhesion to the extracellular matrix, which is mediated by integrins. They reported that lipid rafts are controlled by integrins to target Rho and Rac GTPases to specific plasma membrane domains and to couple them to their downstream effector molecules [53–55].

A detergent-free method was used to demonstrate the localisation of a number of proteins including Ras, the adapter protein Grb2, Erk2 (extracellular signal-regulated kinase 2) and the tyrosine kinases Fyn, TrkB (tyrosine kinase receptor B) and nerve growth factor (NGF) receptor (TrkA) in low buoyant density domains lacking caveolin-1 [56]. Lipid rafts have also been associated with signalling molecules in lymphocytes, which do not contain caveolin. For example, the T-cell receptor has been located in detergent-insoluble rafts following its activation, as have the signalling molecules Shc, Ras, Syk, and the Src kinases Lck and Fyn [32, 57, 58]. It must however be noted that these two studies utilised the detergent extraction method, and it has been shown that proteins associated with rafts and caveolae can be differentially soluble in different detergents [59], possibly due to a difference in their interactions with rafts/caveolae or due to the presence of distinct types of raft [60]. Therefore the detergent extraction method does not give an accurate view of all the proteins present in rafts. Finally, recent studies suggest that the differential localization of rafts is important in the control of cell migration [61, 62].

Table 1 summarises the proteins and lipid species found in lipid rafts and caveolae. This is not an all inclusive list but contains many of the proteins and lipids relevant to this discussion. What causes these proteins to target to lipid rafts and caveolae? Melkonian *et al.* [63] hypothesised that this might depend on the lipid modification of the proteins and their affinity for the liquid-ordered phase. Acyl chains, such as myristate and palmitate, and those found in GPI-anchored proteins, are saturated and likely to partition into liquid-ordered domains, while, for example, prenyl chains are bulkier and less likely to favour this phase. Melkonian *et al.* [63] showed that detergent-insoluble membranes were indeed enriched in palmitoylated proteins, although not all palmi-toylated proteins in the cell were targeted to these domains. In contrast, prenylated proteins were excluded from the domains. In a later study, Zacharias *et al.* [64] produced similar results using a detergent-free method. They used FRET between two variants of green fluorescent protein: cyan fluorescent protein (CFP) and yellow fluorescent protein (YFP), which had been mutated so that they were unable to dimerize. Attaching myristoyl and palmitoyl chains to CFP and YFP caused them to form clusters in the membrane, and this was disrupted by depleting cholesterol. In addition, they were shown to cluster with caveolin. However, while prenylated fluorescent proteins were seen to cluster, this was not disrupted by cholesterol depletion. They also detected acylated, but not prenylated fluorescent proteins in detergent-insoluble fractions. Taken together, the results of these two studies do indicate that acylated proteins are more likely to be targeted to lipid rafts and caveolae; however, the prenyl chain used by Zacharias *et al.* [64] is not truly representative of those found in the cell [65], and so the exclusion of prenylated proteins is not conclusive.

## ROLE OF LIPID RAFTS AND CAVEOLAE IN SIGNALLING BY GROWTH FACTOR RECEPTORS

The role of lipid rafts and caveolae in signalling by several growth factor receptors has been studied, but none more so than the epidermal growth factor receptor (EGFR). We will therefore be paying particular attention to this receptor. However, we will also discuss several other receptors for which information is sparser.

### 1) Epidermal Growth Factor Receptor (EGFR)

#### Structure and Function of the EGFR

The epidermal growth factor receptor (EGFR) signaling pathway is an important mediator of cancer cell oncogenesis, proliferation, maintenance, and survival [66]. This receptor is expressed in all epidermal and stromal cells as well as some glial and smooth muscle cells, and has several ligands, including EGF itself, transforming growth factor (TGF)-α, and heparin-binding EGF (HB-EGF) [66]. It can also be transactivated by the binding of ligands to certain G-protein coupled receptors, such as the angiotensin II receptor [67]. The EGFR has three known homologues: ErbB2 (Neu or HER2), ErbB3 (HER3) and ErbB4 (HER4), with which it can form heterodimers [68]. Ligand binding causes homo- or hetero-dimerization of the receptor and activates its tyrosine kinase activity, allowing it both to autophosphorylate and to phosphorylate downstream signalling molecules [69]. The EGFR consists of a number of extracellular domains forming the ligand-binding site [68]. There is then a transmembrane sequence and juxtamembrane domain, followed by an intracellular kinase domain and C-terminal domain. Five autophosphorylation motifs are found in the C-terminal domain, providing docking sites for proteins containing SH2 (Src homology region 2) and phospho-tyrosine binding (PTB) domains (e.g., Grb2). The C-terminus can also act as an auto-inhibitory domain when not phosphorylated [66]. Nine phosphorylation sites were described in the EGFR, which differentially regulate the activation of downstream signalling pathways [70, 71]. Firstly there is the well-characterised p42/44 mitogen-activated protein kinase (MAPK) pathway. This begins with the recruitment of Grb2, which contains an SH2 domain and can bind either directly to the phosphorylated EGFR, or to Shc, an adapter protein that becomes associated with and tyrosine phosphorylated by the EGFR. Grb2 is associated with SOS, a Ras guanine nucleotide exchange factor, which is then able to activate membrane-associated Ras [68]. Ras is activated by the exchange of its associated GDP with GTP, and in turn activates Raf-1, a serine/threonine kinase, which leads to the activation of the MAPK pathway. This involves the phosphorylation of MEK (MAP kinase kinase) and the activation and translocation of Erk1 and Erk2 to the nucleus, where they phosphorylate transcription factors such as Elk, stimulating cell proliferation and motility.

**Fig. (2):**
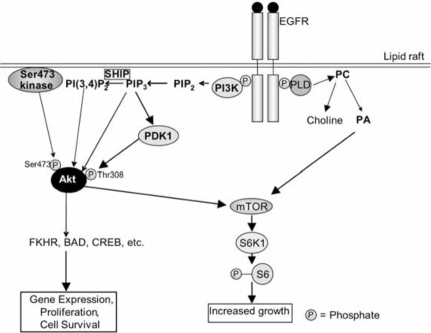
**The EGFR signalling pathway in lipid rafts.** See text for description of the pathway. The PLD shown here is PLD-2, which is activated directly by the EGFR. It must be also emphasised that at least three steps contribute to activation of Akt: (i) binding of PIP_3_/PI(3,4)P_2_ to its Pleckstrin Homology domain; (ii) phosphorylation on Thr308 by PDK1; and (iii) phosphorylation on Ser473. PLD: phospholipase D; PA: phosphatidic acid; PC: phosphatidylcholine; mTOR: mammalian target of rapamycin; EGFR: epidermal growth factor receptor; FKHR: Forkhead Box, subgroup O, transcription factors; CREB: cAMP response element binding protein; PDK1: 3-phosphoinositide-dependent kinase-1; PI3K: phosphatidylinositol 3-kinase; PIP_2_: phosphatidylinositol-4,5-bisphosphate; PI(3,4)P_2_: phosphatidylinositol-3,4-bisphosphate; PIP_3_: phosphatidylinositol-3,4,5,-trisphosphate; S6K1: ribosomal protein S6 kinase 1.

Other targets of the EGFR include phospholipase Cγ (PLCγ), phospholipase D (PLD)-1 and –2, and phosphatidy-linositol-3-kinase (PI3K). PLCγ hydrolyses phosphatidylinositol-4,5-bisphosphate (PIP_2_) to produce diacylglycerol and inositol 1,3,5,-trisphosphate (IP_3_), the latter of which releases calcium from intracellular stores. This results in the activation of many calcium-dependent enzymes and downstream pathways [68]. PLCγ can also mobilise actin modifying proteins, enhancing cell motility [66]. PLD-1 may be activated *via* protein kinase Cα [72], while PLD-2 can be activated directly by the EGFR [68]. PLD hydrolyses phosphatidylcholine to produce choline and phosphatidic acid (PA) (Fig. 2). PA in turn activates mTOR (mammalian target of rapamycin), with which it directly interacts [73]. Among other things, mTOR activates the ribosomal protein S6 kinase 1 (S6K1), which phosphorylates the ribosomal S6 protein, leading to increased cell growth (Fig. 2). PI3K phosphorylates PIP_2_ to produce phosphatidylinositol-3,4,5,-trisphosphate (PIP_3_), which can be converted to phosphatidy-linositol-3,4-bisphosphate (PI(3,4)P_2_) by SH2-containing inositol-5-phosphatase (SHIP) [74, 75] (Fig. 2). PIP_3_ and PI(3,4)P_2_ stimulate the activity the serine/threonine kinase Akt (also known as protein kinase B) by binding to its Pleck-strin Homology (PH) domain. Also required for activation of Akt is its phosphorylation on threonine 308, which occurs *via* phosphoinositide-dependent kinase-1 (PDK1), an enzyme activated by PIP_3_ (Fig. 2). PDK1 can also activate S6K [76]. Phosphorylation on serine 473 is controlled by the ric-tor-mTOR complex [77]. It is of interest to note here that a lipid raft-associated Akt Ser473 kinase was described [78], and subsequently shown to be DNA-dependent protein kinase (DNA-PK) [79]. This enzyme may also be involved in the regulation of Akt under specific conditions. Akt can phosphorylate proteins such as CREB (cAMP response element binding protein), pro-caspase-9, BAD and the forkhead family of transcription factors (FKHR), and is involved in the regulation of apoptosis, gene expression and cell proliferation [74].

Finally, the activation of Src kinases is associated with activation of the EGFR. Src kinases have numerous substrates, including PI3K and elements of the cytoskeleton [68].

#### Role of Lipid Rafts and Caveolae in Signalling by the EGFR

The involvement of lipid rafts and caveolae in signalling by the EGF receptor (EGFR) is by no means clear cut. Indeed, there is a great deal of discrepancy in the literature, due in the most part to the use by some researchers of methods which do not allow exclusive separation of lipid rafts and caveolae. Evidence for EGFR Signalling within Caveolae

##### Evidence for EGFR Signalling within Caveolae

Mineo *et al.* [80] used a detergent-free fractionation method to isolate low buoyant density, caveolin-rich membrane fractions from Rat-1 cells, some of which had been treated with EGF. H-Ras, Grb2 and SOS-1 were concentrated in the caveolin-rich fraction, both in unstimulated cells and in those that had been exposed to EGF. On the other hand, they found that Raf-1 was only present in the caveolin fraction following stimulation by EGF, but that this Raf-1 was active. Conversely, the EGFR was present in the caveolin fraction in unstimulated cells, but its levels began to decline after 30 seconds’ stimulation by EGF, and following 60 minutes’ stimulation it was no longer detectable in this fraction. These results were taken to indicate that EGFRs were present in caveolae, causing them to cluster and therefore allowing efficient dimerization. However, following activation, the EGFR would migrate out of caveolae, allowing termination of the response. Mineo *et al.* [81] obtained similar results for the EGFR, showing that before activation, 60.5% of EGFRs were present in the caveolin fraction of human fibroblasts, but there was a dramatic decrease in number following EGF application.

Jang *et al.* [82] also localised the EGFR in caveolin-rich membrane fractions in unstimulated cells (in this case, A431 and COS-7 cells). Following treatment with EGF, phosphol-ipase Cγ (PLCγ) was recruited to the caveolin fraction. They also used immunostaining of COS-7 cells to visualise the recruitment of PLCγ to the membrane: following EGF stimulation, labelled PLCα was shown to co-localise with caveolin. In addition, depletion of cellular cholesterol resulted in translocation of the EGFR and caveolin-1 out of the low buoyant density fraction and prevented recruitment of PKCγ to this fraction, as well as inhibiting PIP_2_ turnover. However, PLCγ phosphorylation by the EGFR was not decreased upon cholesterol depletion, suggesting that the EGFR can still activate PLCγ when it is delocalised from caveolae. It was therefore hypothesised that loss of PIP_2_ turnover was due to its own delocalisation and not that of the EGFR or PLCγ.

A separate study by Han *et al.* [72] placed another downstream effector of EGFR signalling, phospholipase D1 (PLD1) in the caveolin-rich membrane fraction of COS-7 cells. Stimulation by EGF caused phosphorylation of PLD1 in the caveolin-rich fraction, with kinetics similar to which protein kinase Cα appeared in this fraction. Palmitoylation of PLD1 was required for its location within the caveolin-rich fraction, and for its activation *via* the EGFR, which resulted in the local production of the secondary messenger PA.

#### Evidence that Caveolae Inhibit EGFR Signalling

Several lines of evidence dispute the idea that caveolae are the locations of EGFR signalling, and suggest that caveolae may in fact have a negative regulatory role. More precisely it appears that the scaffolding domain of caveolin-1 stabilizes the EGFR kinase in an inactive conformation [83], and the same negative regulation also exists in the case of the ErbB2 tyrosine kinase activity [84, 85]. Couet *et al*. [83] demonstrated the co-fractionation of the EGFR, and its close relative ErbB2, with caveolin. They went on to show that the EGFR coimmunoprecipitated with caveolin-1, and that the scaffolding domain of caveolin-1 (residues 82-101) could bind to the EGFR *in vitro*. A caveolin-binding motif was identified in the cytoplasmic kinase domain of EGFR, and is conserved in most receptor tyrosine kinases. In addition, the caveolin-1 scaffolding domain dose-dependently inhibited the kinase activity of the EGFR *in vitro*. The interaction was independent of EGFR activity and did not require tyrosine phosphorylation of caveolin-1.

Interestingly, it has been shown that overexpression of ganglioside GM3 causes increased interaction of the EGFR with caveolin-1 and hence inhibits EGFR signalling [40]. Furthermore, Engelman *et al*. [86] showed that overexpression of caveolin-1 in a fibroblast cell line inhibited signal transduction by the EGFR. Caveolin-1 expression also inhibited the activity of Raf-1, MEK-1 and Erk2, and the scaffolding domains of caveolin-1 and caveolin-3, but not that of caveolin-2, could directly inhibit the activity of MEK-1 and Erk2 *in vitro*. In addition, downregulation of caveolin-1 causes hyperactivation of the same kinase cascade [87].

Zhang *et al.* [88] also provided evidence that caveolae inhibit EGF signalling. They observed that motile mammary adenocarcinoma cells failed to express normal levels of caveolin-1, in contrast to their non-motile counterparts. Expression of caveolin-1 in motile cells inhibited EGF-dependent lamellipod extension and migration in the motile cell line. It was also shown to prevent activation of the p42/44 MAP kinase cascade, which is possibly one mechanism by which it blocks migration.

Further evidence of an inhibitory role for caveolae came from a study on senescent cells by Park *et al.* [89]. Senescent cells show a decreased response to EGFR signalling, and the phosphorylation of Erk1/2 upon EGFR activation was delayed in senescent fibroblasts. Furthermore, they found that the levels of both caveolin-1 and caveolin-2 were significantly increased in senescent cells compared with young cells, and that old fibroblasts contained over 10 times more caveolae. They also reported that all the major organs in old rats showed increased numbers of caveolae in their cells, and that the increase in caveolae numbers was accompanied by an increase in cellular cholesterol levels. In artificially aged cells, which did not overexpress caveolin, the response to EGF was not reduced, as it would be in normally aged cells. Moreover, overexpression of caveolin-1 in young cells inhibited Erk1/2 activation upon EGF stimulation. This suggests that caveolin plays a role in the age-related decrease in EGFR signalling, though other factors might also be involved.

##### Evidence for the Involvement of Lipid Rafts in EGFR Signalling

Another line of evidence implicates lipid rafts, and not caveolae, in EGFR signalling. Firstly, Waugh *et al.* [90] showed that, although the EGFR was present in the low buoyant density, caveolin-rich fraction from A431 cells, it could not be co-immunoprecipitated with caveolin, and was soluble in Triton X-100, in contrast to caveolin, which was insoluble. Phosphorylated EGFR also did not co-immunoprecipitate with caveolin-1, arguing against a caveolar location for EGFR signalling.

Roepstorff *et al.* [60] showed that depleting cholesterol from the membrane increased ligand binding to the EGFR. Depletion of cholesterol seemed to have this effect by increasing the number of available receptors in the membrane, although the total number of receptors was not changed [91]. It was also demonstrated that oncogenic EGFRs reside in caveolae even after ligand binding, genereting altered signaling pathways such as the enhanced tyrosine phosphorylation of the caveolar molecules, caveolin-1 and dynamin [81]. Li *et al.* [92] demostrated that the depletion of cholesterol also induces rafts disruption with a consequent Bcl-X_L_ downregulation and Akt inactivation. It is intriguing that EGF administration after caveolae disruption could not restore Akt activation once rafts were disrupted, but cholesterol administration restored Akt activity also in absence of EGF [92, 93].

Roepstorff *et al.* [60] used immunofluorescence to study the relative locations of caveolin and the EGFR on the plasma membrane, and did not find any significant co-localisation. Finally, it was found that although caveolin-1 was insoluble in Triton X-100, the EGFR was soluble in this detergent, but insoluble in another detergent, Brij 58, supporting the idea that it is present in Brij 58-insoluble rafts. It is unknown whether these are a subset of rafts or whether this reflects a certain type of association of the EGFR with rafts [92]. The authors of this paper suggested that sequestration in rafts inhibits ligand-binding to the growth factor receptor, and that depletion of cholesterol allows release of the EGFR from rafts, thereby increasing its activation.

Ringerike *et al.* [94] used immuno-electron microscopy to show that only 7% of EGFRs in A431 cells were localised in caveolae. In contrast to the results reported in [80], they did not find any difference in the distribution of EGFRs following stimulation with EGF. As in Roepstorff *et al*. [60], a co-localisation of the EGFR with markers of lipid rafts was found, and approximately 40% of receptors were present within rafts, which did not change following stimulation with EGF. Depletion of cholesterol increased dimerization of the EGFR, but, in contrast to [60], an increase in EGFR levels at the plasma membrane was observed following cholesterol depletion. Further support came from Ushio-Fukai *et al*. [95], who found that depletion of cholesterol enhanced EGF-mediated autophosphorylation of the EGFR.

Matveev and Smart [96] showed that, although present in the caveolin-rich fraction, only 3% of EGFRs in Swiss 3T3 cells co-immunoprecipitated with caveolin in unstimulated cells, with similar results obtained for cells exposed to EGF for ten minutes. However, the EGFR did co-immunoprecipitate with CD55, a marker of lipid rafts, and was of a molecular weight consistent with tyrosine phosphorylation. Interestingly, in cells stimulated with EGF for 60 minutes, 87-94% of EGFRs co-precipitated with caveolin. At this time, the EGFRs were no longer phosphorylated, which they had been after ten minutes’ treatment with EGF. This suggests that signalling might actually occur in rafts, and that sequestration in caveolae following prolonged exposure to ligand may facilitate receptor desensitisation. This is in agreement with the idea that caveolae inhibit EGFR signalling; however, it contradicts the results of Roepstorff *et al*. [60] and Ringerike *et al.* [94], which suggest that rafts also are inhibitory, and is also in direct contradiction to the results of Mineo *et al*. [80, 81].

The results of Zhuang *et al*. [97] suggested an activatory role for lipid rafts in EGFR signalling. This study used the prostate cancer cell line LNCaP, which was shown to contain Triton X-100-insoluble fractions, but not caveolin-1. It was found that, in unstimulated cells, the EGFR was present in detergent-soluble, but not insoluble membranes. Following stimulation with EGF, phosphorylated EGFR appeared in the detergent-insoluble fraction, but was not detected in the soluble fraction. In addition, treatment with filipin decreased the amount of phosphorylated EGFR in the insoluble fraction following EGF stimulation, but this was restored to normal following the addition of cholesterol. The presence of caveolin also did not alter the levels of Akt activation before cholesterol depletion, suggesting that caveolin-1 might not inhibit this pathway as it does MAPK.

Babuke *et al*. [37] also recently demonstrated a role for reggie-1 in EGFR signaling. After EGFR activation, reggie-1 colocalized with the receptor in the plasma membrane and the endosomes. It was also evident that its Tyr phosphorylation involved Src kinases [98]. A role for reggie-2 in raft-mediated endocytosis has also previously been suggested [99]. These results open the interesting possibility that reggie can also play a role in the raft-mediated endocytosis and signaling of the EGFR [100] which also involves Src kinases [101].

In summary, most evidence indicates an inhibitory role for caveolae in EGFR signalling, which might contribute to the desensitisation of receptors [96]. Furthermore, as Park *et al*. [89] demonstrated that older cells, which have a reduced response to EGF, have elevated numbers of caveolae, it is likely that one way in which cells can control the level of signalling by the EGFR is by regulating their number of caveolae. However, it is possible that caveolae only inhibit certain downstream pathways, such as MAPK, and not others, such as PI3K/Akt. There is a certain amount of disagreement in the data concerning whether lipid rafts play an inhibitory or activatory role in EGFR signalling, in that some researchers reported an increased level of signalling following cholesterol depletion, while others have located activated EGFRs in lipid raft fractions. Given that the cells used by Roepstorff *et al.* [60] and Ringerike *et al*. [94] also contained caveolae, however, and the fact that phosphorylated EGFRs were clearly localised to rafts by Zhuang *et al.* [97] and Mat-veev and Smart [96], it is likely that lipid rafts could play an activatory role in EGFR signalling. The bulk of the evidence suggests that the EGFR is constitutively present in rafts, but that its association with rafts is probably strengthened following activation, causing it to become insoluble in Triton X-100. It could then initiate signalling pathways in these domains, which contain a number of molecules downstream from the receptor.

#### Association of Downstream Signalling Pathways with Rafts

Assuming that EGF signalling is localised to rafts, it is necessary to note that not all the downstream molecules may act in these domains. For example, there is evidence to suggest that activated Ras might act outside of rafts. There are three isoforms of Ras: N-Ras, H-Ras and K-Ras, of which K-Ras is most efficient at activating Raf, while H-Ras efficiently activates PI3K [102]. Fukano *et al.* [103] used immunogold labelling to show that some H-Ras, but not K-Ras, was localised to caveolae, and that non-caveolar H-Ras co-localised with a marker of lipid rafts, while K-Ras did not. However, activated H-Ras (G12V) was almost completely excluded from the caveolae/raft fraction. More recently, Prior *et al.* [104] reported that H-Ras exists in a dynamic equilibrium between lipid rafts and non-raft membrane. However, constitutively active H-Ras (G12V) was not localised to lipid rafts, but to cholesterol-independent microdomains. They also examined the distribution of K-Ras, and found that it was localised to cholesterol-independent microdomains. Together, these results indicate that K-Ras and H-Ras occupy different microdomains, and that activated Ras is not associated with rafts.

Given that activated Ras is excluded from rafts, presumably it activates Raf outside of rafts, and it seems strange that Mineo *et al.* [80] found phosphorylated Raf-1 in rafts, suggesting that it returns to these domains upon activation. However, Chen and Resh [105] created several Raf-1 constructs that localised to rafts to different extents, and the extent of localisation to rafts did not affect the extent of Erk activation, Erk being excluded from rafts. The cholesterol depletion data gave varying results: Peiro *et al*. [106] found that cholesterol depletion did not affect MAPK activation by the EGFR, while Chen and Resh [105] found that MAPK activation was increased, but that this occurred *via* PI3K. They suggest that it is due to factors other than raft disruption. Furuchi and Anderson [107] also detected increased EGF-stimulated Erk activation following cholesterol depletion. These results are complicated by the presence of caveolae, and the recent discorvery of a cholesterol-regulated Erk phosphatase [108]. Therefore the evidence suggests that, although H-Ras is probably activated in rafts initially (given that the EGFR, Grb2, Sos and Shc have been found in rafts [57, 80]), activated Ras moves out of rafts to activate Raf, and a raft location for Raf is not strictly required for activation of Erk. However, as mentioned in below sections, studies of other systems have shown that cholesterol depletion can inhibit the MAPK pathway, suggesting that this may vary depending on the receptor involved.

Alternative pathways are related to Ras activation and can act in balance with the EGFR, one of the most important already described is the presence in lipid rafts of the Spred-1 protein. This protein, recruited to the lipid raft/caveolae, efficiently interacts with Ras and also Raf-1 by interacting *via* caveolin-1, resulting in a strong inhibition of the Ras/ERK pathway [109].

As mentioned above, the activation of PLCγ by EGF signalling is not affected by cholesterol depletion, suggesting that a raft location is also not required for the activation of this pathway [82]. However, this study also showed that PIP_2_ turnover was dependent on rafts. There is also evidence that PLD is activated in rafts: Han *et al.* [72] localised activated PLD in caveolae/raft fractions following stimulation with EGF. In addition, there is evidence that the PI3K/Akt pathway is localised to rafts, as cholesterol depletion prevented EGF-induced phosphorylation of Akt [92]. Furthermore, PIP_2_, a substrate of PI3K, has been found in rafts [110], as has the Akt Ser473 kinase [78]. Hill *et al.* [78] therefore hypothesise that a constitutively active Ser473 kinase is present in rafts, and the activation of PI3K (e.g., by the EGFR) and subsequent generation of PIP_3_ from PIP_2_ results in the recruitment of PDK1 and Akt to rafts.

In summary, most of the published evidence suggests a raft localisation for PLD and the PI3K pathway. In particular, as cholesterol depletion prevents Akt phosphorylation [92], a raft localization may be required for adequate functioning of this pathway. In contrast, Ras acts outside of rafts, and while activated Raf and PLCγ have been found in rafts, they need not be present in rafts in order to signal normally.

Therefore, different pathways downstream from the EGFR might be differentially dependent on lipid rafts for their activation.

### 2) Platelet-derived Growth Factor Receptor (PDGFR)

#### Structure and Function of the PDGFR

There are two types of PDGFR: the α-receptor (PDGFRα) and the β-receptor (PDGFRβ), which bind different PDGF types and have distinct but overlapping cellular distributions [111]. The two receptors have a similar structure, possessing five immunoglobulin-like domains extracel-lularly, and in the intracellular region a juxtamembrane domain, tyrosine kinase domain and C-terminal domain. This is reminiscent of the EGFR; however, a distinguishing feature of the PDGFR is that its kinase domain is split in two by a non-kinase 100 amino acid insert [111, 112]. Upon binding of ligand, the receptor dimerises, and can form either homo- or heterodimers, depending on the isoform of PDGF [112]. As for the EGFR, activation results in autophosphorylation of the receptor, increasing its kinase activity and providing docking sites for proteins with SH2 domains. Downstream molecules activated by the PDGFR include PI3K, PLCγ, several members of the STAT family and the non-receptor tyrosine kinase Src. It can also activate the Ras/MAPK pathway *via* Grb2 and SOS [111]. Activation of the PDGFR provokes several responses: early responses include intracellular Ca^2+^ flux and the reorganisation of the cytoskeleton, resulting in the formation of plasma membrane processes. Later effects include cell migration, proliferation and differentiation [112].

#### Involvement of Lipid Rafts and Caveolae in Signalling by the PDGFR

Again there are a number of conflicting results concerning the role of lipid rafts and caveolae in PDGFR signalling. Some studies suggest that caveolae are the sites of signalling; some suggest that caveolae are inhibitory and that signalling occurs in rafts.

Liu *et al.* [113] localised the PDGFRβ to the low buoyant density, caveolin-rich fraction of human fibroblasts, indicating that it was present in either caveolae or rafts. They then used immunocytochemistry to demonstrate that the PDGFRβ was found in distinct patches on the cell surface, and that it co-localised with caveolin in caveolae-like structures. Following stimulation with PDGF, phosphorylated PDGFRβ was found in the caveolin-rich fraction, as were increased amounts of the tyrosine phosphatase Syp, Shc, and MAPK. However, it is important to note that this fraction will also have contained lipid rafts.

Liu *et al*. [114] first showed by immunoblotting that the PDGFRβ, Ras, Raf-1, MEK-1 and Erk2 were all present in caveolae/raft fractions from unstimulated human fibroblasts. The localization of Erk2 and the PDGFR in caveolae was confirmed using immunocytochemistry. Stimulation with PDGF resulted in accumulation of a number of phosphorylated proteins, including activated MAPK, in the caveolae/raft fraction. Immunogold labelling seemed to confirm the presence of tyrosine-phosphorylated proteins in caveolar structures following but not prior to PDGF treatment. Liu *et al.* [115] used a technique designed to separate caveolae from lipid rafts, and found the PDGFRβ to be associated with these domains, along with molecules known to act downstream of the receptor, such as PLCγ, PI3K and non-receptor tyrosine kinases. In addition, they used confocal immunofluorescence microscopy to show that PDGF treatment caused tyrosine phosphorylation of proteins in caveolae, both in cell culture and in intact rat lungs. Disruption of caveolae by cholesterol depletion prevented PDGF-induced tyrosine phosphorylation; the PDGF receptor itself was phosphorylated, but less so than in the control. On the other hand, tyrosine phosphorylation by vanadate was not reduced by cholesterol depletion. Again it must be noted that cholesterol depletion also affects rafts, and can often give unreliable and conflicting results.

The results related above suggest a caveolar location for PDGF and at least some PDGF-induced tyrosine phosphorylation. However, this does not agree with the observation of Couet *et al*. [83], since the PDGFR contains a motif associated with binding to the scaffolding domain of caveolin, an interaction which is usually inhibitory. Furthermore, Yamamoto *et al.* [116] showed that both the PDGFRα and PDGFRβ, which were present in the caveolae/raft fraction, could be immunoprecipitated with caveolin-1. Moreover, the phosphorylation of PDGFRαs was inhibited in a dose-dependent manner by the scaffolding domains of both caveolin-1 and caveolin-3, but not caveolin-2.

Work by Matveev and Smart [96] also suggested an inhibitory role for caveolae, and localised PDGF signalling to rafts. They showed that, although the PDGFR was located in the caveolin-rich, caveolae/raft fraction, it coimmunoprecipitated with CD55, a marker of non-caveolar rafts, and only 3% co-immunoprecipitated with caveolin. Following treatment with PDGF, the PDGFR that co-immunoprecipitated with CD55 was of a molecular weight consistent with tyrosine phosphorylation; little PDGFR precipitated with caveolin. However, most of the PDGFR co-immunoprecipitated with caveolin after exposure to PDGF. This is similar to the results obtained for the EGFR in the same paper. Indeed, antibodies to the PDGFR precipitated membranes containing the EGFR and vice versa, suggesting that the two receptors are present in the same microdomains. This is supported by the fact that Matveev and Smart [96] showed that pre-treatment of Swiss 3T3 cells with either EGF or PDGF for 60 minutes caused sequestration of the receptor for the other ligand, preventing ligand binding and activation. Filipin and the cholesterol-depleting agent methyl-β-cyclodextrin allowed re-exposure of the same receptors. Pre-treatment with either of these substances also prevented sequestration of either receptor. Sequestration was also prevented in cells lacking caveolin-1, supporting a role for caveolae. This indicates that cross-talk between the PDGFR and EGFR occurs, supporting the hypothesis that they are present in the same microdomains, and implicates caveolae as sites of sequestration.

The results just reported are very difficult to reconcile. The results of Liu *et al.* [113–115] suggest that PDGF signalling occurs in caveolae; however, the more recent study by Matveev and Smart [96] suggests that the PDGFR is normally located in lipid rafts, and re-localises to caveolae after prolonged exposure to ligand. In support of a role for lipid rafts is the cross-talk that occurs between the PDGFR and the EGFR, suggesting that they are present in the same microdomains. As discussed above, most evidence suggests that the EGFR is associated with rafts.

It is possible that some PDGFR is present in lipid rafts and some in caveolae. Although Liu *et al.* [113, 114] demonstrated that the PDGFR was present in caveolae, their immu-nocytochemistry/immunogold labelling do show that it is also present outside of caveolae; as it was detected mainly in caveolae/raft fractions, it is therefore likely to be also present in rafts. In addition, the tyrosine-phosphorylated proteins detected by Liu *et al*. [113, 115] were present in the caveolae/raft fraction and could have been associated with rafts.

In summary, it is not possible to say with certainty what the role of lipid rafts and caveolae is in signalling by the PDGFR, although the location of phosphorylated proteins in the caveola/raft fraction, including MAPK [114] suggests that at least one of these domains is involved in signalling. This is supported by a paper by Mitsuda *et al.* [117], who showed that overexpression of the ganglioside GM1 resulted in the re-location of the PDGFR to non-raft/caveolar membrane and hence an inhibition of PDGFR signalling.

### 3) c-Kit- the Stem Cell Factor Receptor

c-Kit is a type III receptor protein-tyrosine kinase, in a class which also includes the PDGFR, and the CSF-1R. Its extracellular N-terminal domain binds to stem cell factor (SCF) and then induces the dimerization of the receptor resulting in activation of the cytoplasmic catalytic activity. This cytoplasmic domain is needed, together with adaptor proteins such as Grb2 and Grb7, to activate several downstream pathways. One of the most important downstream mediators of c-Kit is PI3K, which then activates Akt, a protein-serine/threonine kinase, promoting cell survival [118, 119]. Other downstream effectors of c-Kit include the Ras/mitogen-activated protein kinases and activators of transcription (Jak/STAT) pathways [120].

Lipid raft clustering was shown after SCF stimulation of hemoatopoietic stem cells (HSCs) and correlated with activation of the Akt-FOXO signalling pathway [121]. Inhibition of lipid raft clustering attenuated SCF signalling and prevented hibernating HSCs from re-entering the cell cycle [121]. Jahn *et al*. [122] showed the translocation of c-Kit to lipid rafts after activation of the receptor by ligand binding. The receptor was then co-localized with signalling mediators such as PDK1 and Akt that transduce the survival signal of SCF/c-Kit [122]. Arcaro *et al*. [110] presented evidence that SCF stimulation induces Src kinase activation in the detergent-insoluble fraction of small cell lung cancer (SCLC) cell lines. Src kinase activity was required for optimal Akt activation, which was supported by the fact that Src and PI3K associated in the lipid rafts fraction of SCLC cells [110]. They also demonstrated that on the other hand, the activation of the Erk pathway after SCF stimulation is independent from the integrity of lipid rafts [110, 123].

### 4) Insulin-like Growth Factor-1 Receptor (IGF-1R)

The IGF-1R binds to and mediates the cellular effects of IGF-1, and also binds with lower affinity to IGF-2 [124]. Like other tyrosine kinase receptors, it contains extracellular ligand-binding and cysteine-rich domains, a transmembrane domain and an intracellular kinase domain. However, it is unusual in existing as a dimer on the cell surface prior to stimulation, and so depends on domain rearrangements rather than dimerisation in order to initiate signalling [124]. Downstream pathways activated by the IGF-1R include the p42/44 MAPK pathway and the PI3K/Akt pathway. The IGF-1R is expressed in a wide range of tissues, and its activation can lead to proliferation, differentation and the prevention of apoptosis [124].

Ravid *et al.* [125] demonstrated that constitutive over-expression of caveolin-1 in MCF-7 breast cancer cells inhibits anoikis by attenuating p53 and p21 activation. From this observation they went further, demonstrating that Akt substrate (pp340) phosphorylation is constitutively present, activating the protein and the signalling through it. The signal through the ERK1/2 pathway was also increased [126]. This study together with others demonstrates that IGF-1R stimulation induces anchorage-independent survival of the cells, through caveolin-1 and Akt, leading to a highly motile phenotype of MCF-7 cells [125, 126].

Podar *et al*. [127] found that depletion of cellular cholesterol prevented recruitment of PI3K to caveolae/rafts by IGF-1, inhibited IGF-1-induced IRS1 (insulin receptor substrate 1 – an adapter protein recruited by the IGF-1 receptor) phosphorylation, and blocked IGF-1-stimulated PI3K and Akt activation, but not Erk phosphorylation. However, cholesterol depletion data are complicated by a number of factors, including the existence of a cholesterol-regulated Erk phosphatase [108], and so are not too reliable. Nevertheless, the fact that Erk was not found in the caveolae/raft fraction indicates that it does not signal in these domains, in support of results reported above.

Maggi *et al.* [128] overexpressed human IGF-1R in mouse fibroblasts, and localised the IGF-1R to the caveolin-rich fraction upon ultracentrifugation in a sucrose gradient. They also showed that IGF-1 stimulation resulted in phosphorylation of caveolin-1 on tyrosine 14. Furthermore, IGF-1 seemed to cause translocation of caveolin-1 from the fraction in which it is normally present (fraction 7) to the lighter fractions 5 and 6, which are thought to contain lipid rafts.

Huo *et al*. [129, 130] also found that IGF-1 was present in the caveolae/raft fraction of 3T3 preadipocytes, and showed by double immunofluorescence staining that the IGF-1R co-localised with caveolin-1α in these cells. Furthermore, the IGF-1R was shown to co-immunoprecipitate and to interact with caveolin-1, and caveolin associated with the activated IGF-1R just as it did with the quiescent receptor. This seems to suggest that caveolin-1 does not, in this case, prevent receptor activation. Because the IGF-1R was present not only in fractions containing caveolin but also in some lighter fractions, Huo *et al*. [129, 130] suggested that it was also present in non-caveolar lipid rafts. It was shown that disrupting lipid rafts and caveolae by cholesterol depletion inhibited IGF-1-induced differentiation of preadipocytes into adipocytes, as well as IGF-1-induced clonal expansion. This was not due to inhibition of receptor activation, but to an inhibition of the downstream molecules Erk1 and Erk2. These results are however in contrast with those of Matthews *et al.* [131], who concluded that rafts were not involved in activation of Erk1/2, and that in general they are not essential components in the transduction of the biological actions of IGF-1.

The results of Huo *et al*. [129], who found that lipid rafts/caveolae are required for IGF-1-induced adipocyte differentiation, are interesting in light of the results of Razani *et al*. [132]. These researchers fed caveolin-1 knockout mice on a high fat diet, and found that these mice were resistant to diet-induced obesity, and showed reduced adiposity as they grew older. As Huo *et al.* [129] point out, this suggests that these mice have abnormal adipocyte differentiation in adulthood, and supports a role for caveolae in adipocyte differentiation, possibly *via* the IGF-1R.

In summary, the data so far suggests that the IGF-1R is associated possibly with both lipid rafts and caveolae, and that one or both of these domains are involved at least in downstream signalling from the receptor. Further work is clearly required to determine whether rafts and caveolae have a distinct role in IGF-1R signalling, and for example to determine the significance of the observed translocation of caveolin-1 into supposed lipid raft fractions following IGF-1 stimulation [128].

### 5 Nerve Growth Factor (NGF) Receptors (TrkA and p75^NTR^)

NGF is a member of the neurotrophin family of growth factors, and can activate two receptors: TrkA and the p75 neurotrophin receptor (p75^NTR^). TrkA is a tyrosine kinase receptor, through which NGF signals cell survival, and p75^NTR^ is a member of the tumour necrosis factor receptor family, which induces cell apoptosis [133]. TrkA has a similar structure and function to the tyrosine kinase receptors discussed above; it can associate with the adapter protein Shc, PI3K and PLCγ, and is capable of activating the Ras/MAPK pathway [134]. The activation of p75^NTR^ may result in the generation of ceramide (*via* sphingomyelin hydrolysis), activation of the JNK (Jun N-terminal kinase) pathway and activation of the transcription factor NF-κB [135].

Bilberback *et al*. [136] demonstrated that p75^NTR^ was present in the caveolae/raft fraction from p75-NIH 3T3 cells, and that it co-immunoprecipitated with caveolin. They also localised p75^NTR^-induced sphingomyelin hydrolysis to this fraction. Wu *et al*. [56] localised TrkA to low buoyant density fractions lacking caveolin, suggesting that it is present in rafts, and Huang *et al*. [137] demonstrated that 40% of TrkA and 60% of p75^NTR^ in 3T3-TrkA-p75 cells was present in the caveolae/raft fraction. p75^NTR^ co-immunoprecipitated with caveolin either with or without NGF treatment; however, the co-immunoprecipitation of TrkA with caveolin could not be demonstrated. Possibly the interaction was of low affinity, as the results of Bilberback *et al*. [136] (see below) suggest that TrkA can interact with caveolin. Cholesterol depletion using filipin altered the distribution of TrkA and inhibited its phosphorylation by NGF.

Huang *et al*. [137] also used PC12 cells, and could not detect caveolin-1 in these cells; however, in the paper dicussed below, Peiro *et al*. [106] did detect caveolin and caveolae in PC12 cells. This issue is therefore controversial. Nevertheless, Huang *et al*. [137] showed that TrkA and p75^NTR^ were present in low buoyant density (caveolae/raft) fractions in these cells in similar proportions to those in 3T3-TrkA-p75^NTR^ cells. Following treatment with NGF, NGF-bound TrkA and p75^NTR^ were detected both in the low buoyant density fractions and other membrane fractions. However, more NGF-bound receptor seemed to be present in the low buoyant density fractions, and these fractions seemed to be enriched in higher affinity receptors, especially with regard to TrkA. Furthermore, activated TrkA was enriched in these fractions compared to other fractions following NGF stimulation. Huang *et al*. [137] then examined downstream molecules of TrkA, and found that, although not present solely in the caveolae/raft fraction, the adapter molecule Shc only co-immunoprecipitated with activated TrkA in this fraction. A similar result was found for PLCγ. This suggests that NGF signalling *via* these molecules at least is localised to caveolae or rafts.

Peiro *et al*. [106] used double immunofluorescence microscopy and immunogold techniques to show that a fraction of TrkA co-localised with caveolin and was present in the caveolae of PC12 cells. Depletion of cholesterol resulted in an increased basal level of MAPK activity, but inhibited the activation of MAPK by TrkA, although not affecting NGF-induced phosphorylation of TrkA. This also suggests an involvement of caveolae and/or lipid rafts in TrkA signalling. In addition, it suggests that the requirement for lipid rafts for MAPK activation may depend on the system studied and receptor involved, as in the same study it was found that EGF-stimulated MAPK activation was not affected by cholesterol depletion. As emphasised above, however, other factors may be involved.

On the other hand, the results of Bilberback *et al.* [136] suggest an inhibitory role for caveolae. They showed that transfection of PC12 cells (which they found to express only very low endogenous levels of caveolin-1) with caveolin-1 suppressed NGF-induced differentiation, which usually results in the development of neurites. In addition, although TrkA was autophosphorylated both in these cells and normal PC12 cells after 15 minutes’ treatment with NGF, after this time TrkA autophosphorylation was rapidly abrogated in cells that had been transfected with caveolin. They also demonstrated that TrkA interacted with caveolin-1, as did p75^NTR^, and an *in vitro* kinase assay indicated that caveolin-1 inhibited NGF-induced TrkA autophosphorylation. The authors conclude that caveolae play a negative regulatory role in TrkA signal transduction.

More recently, additional studies about Trk receptor family (TrkA, TrkB and TrkC) were published, especially regarding the neuronal signaling cascade where these receptors are essential [56, 138]. Limpert *et al*. [139] demonstrated that following NGF stimulation, TrkA is concentrated whithin the lipid raft fraction of the plasma membrane. This is possibly due to the adapter function of CAP, which links TrkA-containing complexes to flottilin. It was also recently demonstrated that there is an interaction of TrkA and TrkB with Src family kinases, such as Fyn and Src [140, 141].

The fact that Bilberback *et al*. [136] found that caveolin inhibits TrkA signalling suggests that rafts might be the more likely location of signalling, similar to the situation with the EGFR. Less information is available for p75^NTR^, but p75^NTR^-induced sphingomyelin hydrolysis at least has been localised to either rafts or caveolae. The results above suggest anyway, that TrkA, and possibly p75^NTR^, are present in both rafts and caveolae. They also suggest that activation of Shc, PLCγ and MAPK by TrkA occurs in either lipid rafts of caveolae.

### 6 Fibroblast Growth Factor Receptor (FGFR)

There are now four known FGFRs, designated FGFR1-4, all of which are tyrosine kinase receptors. FGFR activation can lead to activation of PLCγ, Src kinases and FGF receptor substrate 2 (FRS2). FRS2 in turn recruits Grb2, leading to the activation of the Ras/MAPK pathway, and can also activate protein kinase C [142]. FGF signalling has a number of effects, including the promotion of angiogenesis, wound healing and limb development. Unfortunately, only two studies have been performed concerning the role of lipid rafts and caveolae, as we will summarise below.

Davy *et al*. [143] showed that FGF-2 was able to induce the tyrosine phosphorylation of proteins in the caveola/raft fraction of human neuroblastoma (LAN-1) cells. Some of this phosphorylation required Src kinases, of which Lyn and Fyn were found in the caveolae/raft fraction, and were phosphorylated following FGF-2 application. In addition, a downstream substrate of Src kinases, annexin II, was recruited to the raft fraction upon FGF-2 treatment. The authors noted that FGFR-2 was present in these cells, but that it was soluble in Triton X-100; however, it does not necessarily follow that FGFR-2 was not present in rafts/caveolae. It has been shown, for example for the EGFR [60], that proteins associated with rafts may be differentially soluble in different detergents.

Ridyard and Robbins [138] showed that FGF receptor substrate 2 (FRS2) was present in caveolae/rafts from LAN-1 cells. FRS2 became serine-threonine phosphorylated following FGF-2 stimulation, and this required the activation of Src kinases, PKC, and MEK-1/2, suggesting an involvement of the MAPK cascade. FRS2 was also tyrosine-phosphorylated following FGF-2 treatment. The authors also observed that stimulation with FGF-2 resulted in the recruitment of Grb2, which is downstream of FRS2, to the raft fraction. Enhancing the tyrosine phosphorylation of FRS2 increased the recruitment of Grb2. The fact that FRS2 is myristoylated [144] may contribute to its localisation to rafts.

Both of the above studies suggest that membrane microdomains are involved in FGF signalling and activation of Grb2 and FRS2.

## CONCLUSIONS

Unfortunately, for many of the growth factor receptors discussed, little information is available concerning the role of lipid rafts and caveolae in signalling, and, in addition, much of the data exist is contradictory. This is due in part to the use of methods which do not completely isolate lipid rafts and caveolae, and the use of methods such as cholesterol depletion, which can give unreliable results. Nevertheless, evidence does exist for the involvement of either lipid rafts or caveolae in some aspect of signalling for all the receptors discussed. For caveolae this role seems often, though possibly not always, to be an inhibitory one, due to the inhibitory interactions of caveolae with various proteins, including the EGFR, TrkA, and components of the MAPK pathway. On the other hand, caveolae may not be capable of inhibiting some other downstream pathways, such as PI3K/Akt.

Many studies suggest that membrane microdomains can also act as ‘signalling platforms’ for growth factor receptors. For example, activated growth factor receptors and downstream molecules have been located in caveolae/raft fractions, and in some cases cholesterol depletion has been shown to inhibit growth factor signalling (e.g., see PDGF, IGF-1, NGF). The fact that caveolin-1 inhibits several growth factor receptors and their downstream molecules makes it more likely that rafts in general are the sites of signal initiation. However, it is possible that caveolae may have a role in downstream signalling from the PDGFR, and caveolin has not been shown to inhibit the IGF-1R. An added complication is the fact that only some downstream molecules require rafts to signal: these include the PI3K/Akt and PLD pathways. With regard to the MAPK pathway, the situation seems to be more complicated: activated Ras seems to move out of rafts, but there is disagreement as to whether Erk activation requires rafts. Possibly this depends on the system studied, but the results are complicated by the many effects of cholesterol depletion.

Clearly much further work is required to determine the exact role of rafts and caveolae in signalling from the growth factor receptors mentioned. The EGFR is the only receptor for which quite a large amount of information is available, and the bulk of the evidence suggests that caveolae inhibit signalling, while lipid rafts are likely to act as signalling platforms at least for signalling *via* PLD1 and PI3K/Akt.

## INVOLVEMENT OF LIPID RAFTS AND CAVEOLAE IN MALIGNANT TRANSFORMATION

The fact that lipid rafts and caveolae have been implicated in signalling by growth factor receptors, which are involved in the regulation of proliferation, differentiation, apoptosis and cell migration, suggests the possibility that the alteration of these domains could be involved in malignant transformation. So far, most of the evidence for this theory relates to caveolae, and, as we will describe, caveolin-1 has been identified as a possible tumour suppressor gene. Firstly, however, we will discuss a possible role for lipid rafts in cancer.

### 1 Lipid Rafts

The central role that lipid rafts play in cancer development and metastasis has become evident only in the recent few years. Lipid rafts are areas where many growth factor receptors have been shown to localize, and thus it was obvious to think of these domains as a possible location where cell signalling events can be altered. Some reports such as Liu *et al*. [145] have provided clear evidence that disruption of rafts inhibits EGF-induced chemotaxis of human breast cancer cells. Further investigations also revealed impared directional migration of cells, EGF-induced cell adhesion, actin polymerization and also translocation of some receptors.

Upregulation of the PI3K pathway in cancer often occurs due to a defect in the *PTEN* (phosphatase and tensin homologue deleted on chromosome ten) tumour suppressor gene, which is mutated in numerous cancers [146]. PTEN dephosphorylates phosphatidylinositol phosphates (e.g., PIP_3_, PI(3,4)P_2_), and when this activity is lost it can lead to constitutive activation of Akt, and increased cancer cell survival and proliferation. Moreover, the *PIK3CA* gene, encoding the catalytic p110α isoform of PI3K is mutated in a variety of human cancers [147, 148]. These mutations constitutively activate the catalytic activity of PI3K and are oncogenic [149–151]. Consistent with this, Zhuang *et al*. [97] reported that an inhibitor of PI3K induced apoptosis of LNCaP cells, while the effect of this drug was reversed by treatment with EGF. They localised the activated EGFR to rafts, and treatment with filipin induced apoptosis to a similar extent as the PI3K inhibitor, and under these conditions EGF pre-treatment could not prevent apoptosis. It was shown that filipin prevented constitutive and EGF-stimulated phosphorylation of Akt1, explaining its ability to induce apoptosis and prevent EGF-mediated survival. The authors interpret these results to mean that Akt signalling in these cells is still dependent on upstream signalling from the EGFR, and that EGFR signalling through Akt is dependent upon cholesterol-rich lipid rafts. Transfecting cells with caveolin-1 did not change the effects of cholesterol depletion on EGFR/Akt signalling, suggesting that caveolae are not involved, and furthermore that lipid rafts have a similar effect in cells that do possess caveolae.

The results of Zhuang *et al*. [97] are interesting in the light of the fact that a link has been found between high fat intake and risk of prostate cancer [152–154], and Zhuang *et al.* [97] suggest that this might be related to high cholesterol levels. While depleting cholesterol disrupts rafts and decreases EGFR signalling, increasing cholesterol levels increases the size of rafts [155] and possibly raft number.

Other receptors that are known to accumulate in lipid rafts are involved in the process of malignant transformation. Recently several authors have also focalized on the IGF-1R [156–158]. In the case of this receptor, most of the the evidence seems to go into the direction of alterations in the PI3K/Akt pathway. Constitutively active TrkA mutants can also cause cellular transformation (e.g., in colon carcinoma or acute myeloid leukaemia) [159], and the PDGFR has been observed to be up-regulated or mutated in cancer [111]. Rafts also contain a number of other proteins that can cause cancer if mutated or misregulated, including PI3K, Ras, and Src. While it has been shown that constitutively active H-Ras is excluded from rafts [160], rafts could act to stabilise signalling from other oncogenic proteins like those just mentioned. Furthermore, if cholesterol levels were increased in cells transformed by these proteins, this could stimulate their signalling still further, exacerbating the problem. The recent finding that lipid rafts are the place where all these altered growth factor receptors initiate their signals, suggests that lipid rafts could be the target for new drug development.

Therefore Zhuang *et al.* [97] suggest that cholesterol depletion could be a useful treatment for prostate cancer, and refer to a study by Gordon and Schaffner [161], who found that sterol-binding agents such as filipin reduced prostate gland hyperplasia in dogs with no toxicity. In support of the use of cholesterol depletion for cancer treatment are the results of Podar *et al*. [127]. Cholesterol depletion inhibited IGF-1-induced activation of PI3K and Akt in multiple myeloma cells, and induced G_1_ growth arrest. Given that mutation in *PTEN* and *PIKCA* are commonly observed in human cancer [146, 147], the role of lipid rafts in activation of the PI3K/Akt pathway may be important in numerous malignancies. However, there is still the possibility of cholesterol depletion agents causing toxicity in humans, and *in vivo* studies of tumours are required to confirm the work of Zhuang *et al*. [97] regarding the link between high cholesterol, elevated signalling through the EGFR, and prostate cancer incidence/progression. Intriguingly, caveolin-1 is often up-regulated in prostate cancer cells [162], and its expression is known to be increased by high levels of cholesterol [22]. High cholesterol could therefore be the reason for this. However, as explained below, in other cell types caveolin-1 can act as a tumour suppressor, and cholesterol depletion has been shown to decrease caveolin-1 levels [23], so this treatment might have deleterious effects in these types of cancer and would have to be used specifically.

Several already known anti-cancer agents have recently been found to inhibit tumor cells proliferation, or spreading through the disruption of lipid rafts components [163]. For instance, Huang *et al*. [163] have recently discoverd that Emodi, the major active component of *Rheum palmotum* L., with known anti-cancer activities, is able to induce a significant decrease in cholesterol and sphingolipids in the raft fraction. Data from their study proved that Emodin, through the impairement of lipid raft-associated integrin signaling pathways inhibits adhesion and spreading of tumor cells. Similar results were found using different tumor types [164, 165] and other drugs [166–168].

### 2 Caveolae

The fact that caveolin-1 is capable of suppressing the activity of many components of growth factor signalling pathways (e.g., the EGFR and components of the p42/44 MAPK pathway [86, 88]), makes it an attractive candidate tumour suppressor gene, and several lines of evidence support this proposal.

#### Caveolin-1 is Down-Regulated in Tumours and Tumour Cell Lines

Caveolin-1 has been observed to be down-regulated in a number of tumours and tumour cell lines. Firstly, Koleske *et al*. [169] showed that caveolin-1 mRNA was down-regulated in NIH 3T3 fibroblasts that had been transformed by a number of different oncogenes including v-Abl and H-Ras (G12V). Transformation of cells by v-Abl occurs at least partly *via* the Ras/MAPK pathway [170]. The transformed cells were examined under the electron microscope and observed to contain few if any morphologically identifiable caveolae. In addition, Roussel *et al*. [171] found that very low levels of caveolin-1 were present in several lung cancer cells lines, although in most cases caveolin-2 was present at normal levels [172]. Caveolin-2 was shown to be unable to form caveolae in the absence of caveolin-1 [13], and in this case is also not transported out of the Golgi. The real function of caveolin-2 is still unknown but Webley *et al*. [173] suggest the evidence of its role as associated with the inclusions of obligate intracellular pathogens.

Cassoni *et al.* [174] also demonstrated an evident association between tumor progression and a more structured membranous pattern of caveolin-1 expression. This evidence appears in brain tumor cells derived from 64 patients [174]. Different groups found caveolin-1 to be down-regulated in tumours derived from the ovary, breast and colon [162, 175]. In ovarian carcinoma cell lines this down-regulation required DNA methylation, and transfection of ovarian cancer cells with caveolin-1 decreased cell survival. While two carcinoma cell lines did not down-regulate caveolin-1, they found the caveolin-1 in these cells to be phosphorylated on tyrosine 14. Phosphorylation of caveolin-1 on tyrosine 14 (e.g., by Src) has been shown to stimulate anchorage-independent growth and cell migration [176]. In addition, Wiechen *et al*. [162] found that caveolin-1 was down-regulated in numerous human sarcomas, and interestingly found that treatment with an inhibitor of MEK potently up-regulated the expression of caveolin-1α in a fibrosarcoma cell line, while in this case interfering with methylation had little effect.

#### Re-Expression of Caveolin-1 Inhibits Anchorage-Independent Growth

Engelman *et al.* [177] expressed caveolin-1 in v-Abl- and H-Ras (G12V)-transformed NIH 3T3 cells (which normally lack caveolin), and found that this abrogated the growth of these cells in soft agar. In v-Abl-transformed cells, caveolin-1 expression also led to visible cell death. They also discovered that expression of caveolin-1 inhibited H-Ras (G12V) and MAPK-dependent activation of the c-fos promoter (as determined by expression of a reporter gene attached to the promoter). Furthermore, transfection of F11 cells with caveolin-1 lead to nucleosomal DNA fragmentation, a hallmark of apoptotic cell death. They suggest that re-expression of caveolin-1 inhibits the Ras/MAPK pathway and therefore inhibits anchorage-independent growth, and in the case of v-Abl-transformed cells also causes cell death when colonies reach a certain size.

Zhang *et al*. [88] found that motile mammary adenocarcinoma cells possessed reduced levels of caveolin-1, and that expression of caveolin-1 in these cells using an adenovirus vector blocked anchorage-independent growth. It also blocked EGF-stimulated Erk activation, lamellipod extension and cell migration. In a study by Fiucci *et al.* [178], transfecting MCF-7 human breast carcinoma cells (which usually lack caveolin-1) with wild-type caveolin-1 inhibited proliferation, anchorage-independent growth and invasion of the extracellular matrix. It also prevented activation of Erk1/2 on contact with laminin. Lee *et al.* [179] found caveolin-1 to be down-regulated in several breast cancer cell lines, and found that re-expression of caveolin-1 inhibited growth. Finally, Bender *et al.* [180] demonstrated that re-expression of caveolin-1 in colon carcinoma cell lines in most cases reduced their ability to form tumours in nude mice. However, in 30% of cases tumour formation was not reduced, and in these cases it was found that tumour formation resulted in the selection of cells that did not contain caveolin-1.

#### Targeted Down-Regulation of Caveolin-1 Promotes Cellular Transformation

Galbiati *et al.* [87] used caveolin-1 antisense cDNA to down-regulate caveolin-1 in NIH 3T3 cells. These cells displayed altered morphology, anchorage-independent growth and loss of contact inhibition, all hallmarks of transformation, and were also capable of forming tumours in immuno-deficient mice. It was shown that components of the p42/44 MAPK cascade (MEK and Erk) were constitutively active in these cells, and this was necessary for maintenance of the transformed phenotype. In contrast, the p38 MAPK and JNK cascades were not affected. Jasmin *et al*. found that mechanical down-regulation of caveolin-1 in ischemic brain induce impaired angiogenesis and increased apoptotic cell death, whereas the phenotype of caveolin-2 absence was comparable to the wild-type [181].

#### Caveolin Knockout Mice

Caveolin-1 knockout mice were first reported in 2001, and a number of observations regarding these mice support a role for caveolae in suppressing growth. Razani *et al*. [182] found that embryonic fibroblasts derived from the knockouts proliferated twice as fast as the wild type cells, but this was not due to hyperactivation of the MAPK cascade. Zhao *et al.* also examined knockout mice and noted that they are completeli devoid of caveolae [183]. With the major transcytotic organelle absent, these mice were also predicted to have decreased vascular permeability [183, 184]. The further report of an increase in vascular permeability from Schubert *et al*. was therefore quite surprising [185].

Lin *et al.* [186] also presented evidence that in caveolin-1 null mice there is an increased tumor permeability (defined by the extravasation of Evans blue and deposition of fibrinogen). Tumor permeability and angiogenesis are most likely interdependent, creating a positive feedback to support increased tumor growth. The authors observed a hyper-phosphorylation of VEGFR-2 that could be responsible for the increase in tumor permeability and angiogenesis through activation of several pathways.

Cohen *et al*. [187] examined the hearts of caveolin-1 knockouts and found that they possessed significantly thicker left ventricular walls than normal mice, as well as being significantly heavier overall. Closer examination revealed cardiac myocyte hypertrophy (overgrowth) and fibrosis (formation of fibrous tissue). Erk1/2 was hyperactivated in heart tissue and in isolated cardiac fibroblasts. The levels of cyclin D1 (a regulator of the cell cycle) were not altered, although elevated levels of inducible and endothelial nitric oxide synthase may have contributed to the cardiac hypertrophy. In contrast, hyperactivation of Erk1/2 was not detected in caveolin-2 knockout mice.

Lee *et al.* [176] also examined caveolin-1 knockout mice, and found that although these mice failed to spontaneously develop mammary tumours, their mammary glands had an epithelial cell layer several cells thick. In addition, Capozza *et al.* [188] found that caveolin-1 knockout mice were more susceptible to skin carcinogenesis induced by a known carcinogen, 7,12-dimethylbenzanthracene (DMBA).

Williams *et al.* [189] interbred caveolin-1 knockout mice with tumour-prone transgenic mice that normally develop multifocal mammary lesions. In tumour prone mice lacking caveolin-1, the frequency and size of the lesions were greatly increased, while this was not true for mice heterozygous for caveolin-1. However, in this case it was found that cyclin D1 was up-regulated in the mammary lesions of the caveolin-1 null mice as compared to the normal tumour prone mice, but no change in the activation state of the p42/44 MAPK cascade was observed.

Regarding caveolin-2 there are not really clear evidences about a possible role that it plays in cancer. In fact, lack of caveolin-2 does not affect the presence of caveolae, the expression of caveolin-1 and therefore of the receptors involved in caveolae. But still Schubert *et al*. had evidence about age-related skeletal muscle abnormalities [190].

Regarding caveolin-3 knock-out mice, the only evident phenotype is a disfunction in heart. Woodman *et al.* [191] had evidence of hyperactivation of p42/p44 MAPK (Erk1/2) that plays an important role as an effector of the cardiac hypertrophic response. A cardiomyopathic phenotype was also present in the case of caveolin-1 and caveolin-3 double knock-out mice [192].

#### How is the Expression of Caveolin-1 Down-Regulated in Transformed Cells?

Several studies have investigated caveolin-1 down-regulation in cancer, and hence provided further evidence that it acts as a tumour suppressor. Firstly, Engelman *et al*. [193] showed that caveolin-1 and caveolin-2 are localised in the q31.1 region of chromosome 7, a region that is commonly deleted in human cancers. In addition, caveolin-1 mutations have now been detected: Hayashi *et al*. [194] identified heterozygous mutations in codon 132 (P132L) of caveolin-1 in 16% of human primary breast tumours examined, while failing to observe this mutation in test subjects. Cancers harbouring the mutation were mainly invasive cancers. NIH 3T3 cells transfected with the caveolin-1 mutant showed disruption of the actin cytoskeleton and abnormal morphology, increased growth on soft agar, and constitutive activation of MAP kinases. Transfected cells also demonstrated higher motility and invasive capacity than parental cells or cells transfected with wild type caveolin-1. Lee *et al.* [195] examined the P132L mutation further, and found that, in cells transfected with the mutant protein, the mutant was excluded from low density, Triton X-100 insoluble fractions. Immunofluorescence studies showed that the mutant caveolin-1 was in fact retained at the Golgi complex. They also showed that the mutant protein acts in a dominant negative manner, causing wild-type protein to also be retained at the Golgi complex; this is an important observation, as the mutation was generally found to be heterozygous in breast cancer cells [194].

Hypermethylation of the caveolin-1 promoter may also be a mechanism by which its expression is altered in cancer. Methylation of CpG islands in the promoters of tumour suppressor genes has been observed in transformed cells [196]. Engelman *et al.* found that CpG islands in the caveolin-1 promoter were hypermethylated in two breast cancer cell lines that do not express caveolin-1. Cui *et al.* [197] also noted that caveolin-1 was hypermethylated at CpG islands in its promoter region in prostate cancer cells. However as yet it has not been proven that this methylation leads to down-regulation of caveolin-1, and in fact some of the cancer cells studied by Cui *et al.* [197] actually had elevated levels of caveolin-1. Nevertheless, Wiechen *et al.* [198] did observe that the down-regulation of caveolin-1 in two ovarian cancer cell lines required DNA methylation.

Engelman *et al.* [199] found that caveolin-1 was down-regulated in cells transformed by mutationally activated Ras or Raf, and that treatment with a MEK inhibitor restored caveolin-1 mRNA and protein levels. They suggest therefore that caveolin-1 can be down-regulated by activation of the Ras/MAPK cascade. However, they also found that there were MAPK-independent pathways that could act to down-regulate caveolin-1, for example in v-Src transformed cells. Control of expression appeared to be at the transcriptional level; indeed, transient transfection of CHO cells with activated Raf or Erk down-regulated caveolin-1 promoter activity. Overexpression of protein kinase A was also capable of down-regulating caveolin-1 expression. These results suggest that caveolin-1 can be down-regulated by an oncogenic stimulus such as hyperactivation of the MAPK cascade. Engelman *et al.* [177] also demonstrated that caveolin-1 levels could be regulated by an oncogenic stimulus: they used an NIH 3T3 cell line expressing a temperature-sensitive form of v-Abl. When the kinase was active, caveolin-1 was down-regulated, but at non-permissive temperatures, when the kinase was inactive, caveolin-1 levels returned to those seen in normal cells. They also found that treatment with an inhibitor of MEK led to up-regulation of caveolin-1 (inhibitors of the p38 MAPK pathway, which involves different components, did not affect caveolin-1 levels). Treatment with PDGF or FGF has also been shown to down-regulate caveolin-1 expression in NIH 3T3 cells, and in the latter case at least this was at least partly due to activation of the p42/44 MAPK cascade [87].

Engelman *et al.* [84] examined the relationship between ErbB2 (Neu, HER2) tyrosine kinase activity and caveolin-1 expression. As mentioned previously, the gene encoding ErbB2 (*Neu* or c-*erbB2*) is a proto-oncogene, the overex-pression of which can cause cancer in humans [200]. In addition, mutationally activated forms of ErbB2 have been identified in rats and mice: e.g., oncogenic NeuT, the rat homologue of ErbB2, has an activating mutation in the transmembrane region [200]. Engelman *et al.* [84] transfected fibroblast cell lines with various mutated forms of ErbB2 (including NeuT) that are known to have oncogenic potential. Caveolin-1 mRNA and protein were down-regulated in these cells, but not in cells overexpressing wild-type ErbB2. The down-regulation of caveolin-1 by mutationally activated ErbB2 was partly dependent on the activation of the p42/44 MAPK pathway by the receptor. In addition, caveolin-1 inhibited signal transduction from overexpressed wild-type ErbB2 and NeuT, and this inhibition was mediated by the scaffolding domain. In mammary tumours induced in mice by targeted overexpression of ErbB2, caveolin-1 was also down-regulated, as it was in tumours induced by overexpression of the downstream molecules Ras and Src. These results indicate a reciprocal interaction between ErbB2 and caveolin-1.

#### Caveolin-1 Levels are Not Always Reduced in Transformed Cells

In some tumours and tumour cell lines, it has been found that either caveolin-1 levels are unchanged compared to normal cells, or they are actually increased. For example, while Yang *et al*. [201] found caveolin-1 mRNA levels to be reduced in transformed NIH 3T3 cells, consistent with results discussed above, they also reported elevated caveolin-1 mRNA and protein levels in prostate cancer cell lines derived from metastases. The same was true for tissue samples: normal prostate expressed very low levels of caveolin-1, which was up-regulated in primary tumours and further up-regulated in metastatic tumours. Increased caveolin-1 expression was also detected in breast carcinoma tissue samples. In addition, Ho *et al*. [202] found that while caveolin-1 levels were low in poorly invasive lung adenocarcinoma cells, caveolin-1 was abundant in highly invasive cells. Caveolin-1 levels were also low in tissue samples from primary lung adenocarcinomas, while caveolin-1 levels were generally higher in metastatic tumours. Furthermore, introduction of caveolin-1 into poorly invasive cells induced filo-podia formation and increased the invasive capacity of the cells. Taken together, these results suggest that high caveolin-1 levels correlate with metastasis; this is also consistent with the findings of Bender *et al.* [180], who found that caveolin-1 was up-regulated in a metastatic cell line compared to the parental cell line.

Lavie *et al.* [203] discovered that caveolin-1 was up-regulated in multidrug-resistant colon adenocarcinoma and breast carcinoma cells compared to the parental cell lines. The glycosphingolipid glucosylceramide, a constituent of caveolae, was also up-regulated in multidrug-resistant cell lines, as was caveolin-2. Consistent with these observations, the number of caveolae was increased in multidrug-resistant cells. The authors therefore suggest that caveolae play a role in the development of the multidrug-resistant phenotype. Consistent with results reported above, however, these cells were shown to have a reduced rate of proliferation.

Further results include those of Wiechen *et al*. [198], who also found caveolin-1 to be up-regulated in 11 out of 15 tumour samples from the kidney, prostate and stomach, while Wiechen *et al*. [162] found caveolin-1 levels to be high in both normal mesenchymal tissues and benign mesenchymal tumours. Caveolin-1 has also been shown to be present in T-cell leukaemia cell lines, though not present in normal T-cells [204], and Hurlstone *et al.* [205] found caveolin-1 to be expressed in tumours derived from breast myoepithelium, as well as in normal epithelium. As discussed above, Podar *et al*. [127] found that caveolin-1 was expressed in multiple myeloma cells, and suggest that caveolae have a role in IGF-1R signalling in these cells. However, their experiments do not distinguish between lipid rafts and caveolae.

Interestingly, although Fiucci *et al*. [178] found that expression of caveolin-1 in MCF-7 cells in general inhibited growth and invasion of the cancer cells, these cells were also resistant to anoikis (apoptosis induced by detachment from the extracellular matrix). Based on this observation, they suggest that where cancer cells express caveolin-1, they may have been positively selected due to its negative regulatory effects on apoptosis.

**Fig. (3):**
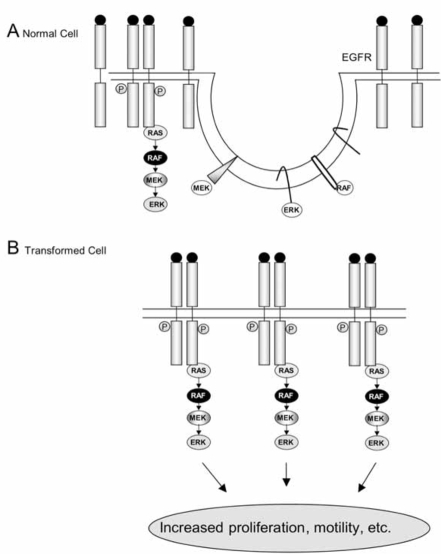
**Model for the role of caveolae in cellular transformation. A**) In a normal cell, caveolin (hairpin structures) binds and inhibits receptors e.g., the EGFR and components of the MAPK cascade. **B**) In a transformed cell with low caveolin levels, more growth factor receptors and components of the MAPK cascade are free from the inhibitory effects, and this leads to increased proliferation. For clarity, not all components of the MAPK cascade are shown. Inactive molecules are shown as open ovals, whereas active molecules are depicted as filled ovals; EGF = epidermal growth factor; Erk = extracellular signal-regulated kinase.

In summary, there is quite a large amount of evidence supporting a role for caveolin-1 as a tumour suppressor gene, including its location at a site commonly deleted in cancers, the identification of mutations in the caveolin-1 gene in some cancers, and its down-regulation in many tumours and tumour cell lines. It could be argued that the use of cell culture systems in many of the papers discussed makes the results less accurate, as conditions used in culture, such as the availability of cholesterol, could affect caveolin-1 expression. However, caveolin-1 has also been found to be down-regulated in tumours themselves, and several independent studies have shown that the re-expression of caveolin-1 in transformed cells inhibits features of the transformed phenotype, such as anchorage-independent growth. Caveolin-1 knockout mice also support the hypothesis, as they display hyperproliferative disorders and hyperactivation of the MAPK cascade, and are at greater risk from carcinogen-induced tumorigenesis [188]. Although these mice do not spontaneously form tumours, knockouts of important cell cycle control agents, such as the cyclin-dependent kinase inhibitor p21, also do not form tumours [206]. Possibly compensatory proteins are involved, but it does not seem that caveolin-3 is one of these, as Razani *et al.* [182] found its levels unchanged in caveolin-1 knockouts. Fig. (3) illustrates how loss of caveolin-1 could lead to cellular transformation: caveolin-1 usually inhibits components of the MAPK cascade and growth factor receptors, and so its loss leads to an increased availability of receptors and signalling molecules, causing hyperproliferation. However, although there is evidence that this pathway plays a role in tumour suppressor activity [87] caveolin-1 also has other effects on the cell cycle. For example, it has been shown to repress the transcription of cyclin D1, which is required for cell cycle progression [207], and increased cyclin D1 expression was shown to be the main reason for the increased tumour formation in tumour prone caveolin-1 knockout mice [189]. In addition, caveolin-1 has been shown to cause cell cycle arrest at the G_0_/G_1_ phase *via* a p53/p21-dependent mechanism [208]. Therefore it is likely that multiple functions contribute to the role of caveolin-1 as a tumour suppressor gene.

The ability of caveolin-1 to negatively regulate growth factor signalling pathways and cell cycle progression, and its identification as a candidate tumour suppressor, might suggest the possibility of cancer treatments based on caveolin-1 or its scaffolding domain. However, the fact that caveolin-1 is up-regulated in some cancers, and especially in metastatic and multi-drug resistant cells, implies that caution would be required here, and clearly further work is required to enable a full understanding as to why caveolin-1 has these effects, and why it is down-regulated in some cancer cells and not others.

## CONCLUSIONS AND FUTURE PERSPECTIVES

The evidence presented in this article supports a role for lipid rafts and caveolae in the regulation of signalling from growth factor receptors. For many of the receptors it is not yet possible to say what the exact roles of each domain are, and further work is required to give a better understanding of the situation. However, caveolae are capable of inhibiting several receptors including the EGFR, TrkA and PDGFR, as well as components of the MAPK pathway (although possibly not the PI3K/Akt pathway). In keeping with this, caveolin-1 seems to act as a tumour suppressor in some cell types. In other cells, however, it is actually up-regulated upon transformation, and it also appears to be re-expressed in me-tastatic tumours. This suggests that other factors are involved, some of which are cell type specific.

A model was presented here to suggest that lipid rafts have an activatory role in EGFR signalling, forming signalling platforms that allow efficient signal initiation and propagation. Such a role for lipid rafts is also possible in signalling by other receptors. Furthermore, this indicates a possible role for lipid rafts in cancer, and it has been suggested that this might be the basis of a link between cholesterol intake and prostate cancer; i.e. that increased cholesterol increases raft size, stabilises EGFR signalling and increases cell growth and survival.

The proposed involvement of lipid rafts and caveolae in cancer suggests the possibility of treatments that target these domains. However, a greater understanding of the reasons why caveolin-1 is up-regulated in some metastatic and drug-resistant cells, and why it only acts as a tumour suppressor in some cancers, is first required. This would be necessary before the development of any treatment based on caveolin-1 or its scaffolding domain, which inhibits components of the MAPK cascade. If such treatments were found to be possible, they would be very specific for certain cancers. The same is true for any attempt to treat cancer by cholesterol depletion, and in this case *in vivo* studies are needed to determine the exact link between cholesterol levels and cancer, and to confirm the hypothesis that increased cholesterol in the plasma membrane increases EGFR signalling.

It is clear to see that reports regarding the protein composition of lipid rafts and caveolae can often be contradictory. In many cases this is due in part to the fact that it is difficult to isolate these domains separately. In addition, there is the problem of detergent insolubility. In some cases, for example for the EGFR, proteins are found to be soluble in one detergent such as Triton X-100, and insoluble in another such as Brij 58. As yet, the reasons for this are unclear: as explained above, it has been suggested that it may reflect a certain type of association of a protein with rafts, or possibly may indicate the presence of distinct types of lipid raft. The latter possibility is intriguing, as it could be that different types of domain mediate signal transduction from different receptors, and this is worth further investigation. Regarding different types of caveolae, two forms, ‘deep’ and ‘shallow’ have been identified, as explained in the introductory section, and again more work is required to determine if they have functional differences.

In the future, experiments on lipid rafts and caveolae will be aided by the use of techniques which allow a more accurate study of these domains [35]. At present, some of the major methods used include cholesterol depletion, which can have many effects on a cell, and the use of detergents, which have unpredictable effects on certain proteins. Because of their small size and their nature as integral components of cell membranes, lipid rafts and caveolae are still difficult to study, and this has been a hindrance to the study of their functions in signal transduction.

## Figures and Tables

**Table 1:** Lipids and Proteins Targeted to Lipid Rafts and Caveolae

Lipid Rafts	Caveolae	Ref.
**Lipid components:**	**Lipid components:**	**[90, 115, 209, 210, 211, 212]**
Cholesterol	Cholesterol	
Sphingomyelin	Sphingomyelin	
Glyco-sphingolipid	Glyco-sphingolipid	
Phosphatidylinositol 4,5-bisphosphate	Phosphatidylinositol 4,5-bisphosphate	
Ganglioside GM1	Ganglioside GM1	
Ganglioside GM3		
**Structural proteins:**	**Signalling proteins:**	
Flotillin-1 and –2	Flotillin-1 and –2	[9, 10, 11, 24, 213]
Caveolin-1, -2 and –3
**Structural proteins:** Flotillin-1 and -2	**Signalling proteins:**	
H-Ras	H-Ras	[32, 42, 48, 49, 56, 57, 115, 160, 214]
G_i_, G_o_, G_β_	G_q_	
Src kinases	Src kinases	
Syk kinase	eNOS	
Grb2, Erk2	Phosphatidylinositol 3-kinase	
Shc	Phospholipase C	
**Receptors:**	**Receptors:**	
PDGF receptor	PDGF receptor	[56, 96, 115, 128, 136, 137, 215]
EGF receptor	EGF receptor	
IGF-1 receptor	IGF-1 receptor
TrkA, TrkB	TrkA	
	CD36	
**GPI-Anchored proteins:**		
e.g. CD59	uPAR	[216, 217]

EGF: epidermal growth factor; eNOS: endothelial nitric oxide synthase; Erk: extracellular signal-regulated kinase; IGF: insulin-like growth factor; PDGF: platelet-derived growth factor; Trk: tyrosine kinase receptor; uPAR: urokinase-type plasminogen activator receptor.

## ABBREVIATIONS

CREB: cAMP response element-binding protein
DMBA: 7,12-dimethylbenzanthracene
EGFR: Epidermal growth factor receptor
Erk: Extracellular signal-regulated kinase
FGF: Fibroblast growth factor
FRET: Fluorescence resonance energy transfer
FRS2: Fibroblast growth factor receptor substrate 2
GPI: Glycosylphosphatidylinositol
IGF-1R: Insulin-like growth factor-1 receptor
IP_3_: Inositol 1,3,5-trisphosphate
JNK: c-Jun N-terminal kinase
MAPK: Mitogen-activated protein kinase
MEK: Mitogen-activated Erk kinase
mTOR: Mammalian target of rapamycin
NGF: Nerve growth factor
PA: Phosphatidic acid
PDGFR: Platelet-derived growth factor receptor
PDK1: 3-phosphoinositide-dependent kinase-1
PI3K: Phosphatidylinostol 3-kinase
PIP_2_: Phosphatidylinositol-4,5-bisphosphate
PI(3,4)P_2_: Phosphatidylinositol-3,4-bisphosphate
PIP_3_: Phosphatidylinositol-3,4,5,-trisphosphate
PKC: Protein kinase C
PLC: Phospholipase C
PLD: Phospholipase D
S6K1: Ribosomal protein S6 kinase 1
SH2: Src homology region 2
SHIP: SH2-containing inositol-5-phosphatase
Trk: Tyrosine kinase receptor
uPAR: Urokinase-type plasminogen activator receptor
